# Letter to the editor regarding ‘a deep learning approach for gastroscopic manifestation recognition based on Kyoto Gastritis Score’

**DOI:** 10.1080/07853890.2025.2552928

**Published:** 2025-09-08

**Authors:** Mengyuan Tang, Wenliang Lv

**Affiliations:** School of Traditional Chinese Medicine, Hubei University of Chinese Medicine, Wuhan, Hubei Province, China

Dear Editor

I read with interest the article ‘A deep learning approach for gastroscopic manifestation recognition based on Kyoto Gastritis Score’[1], which presents a promising AI approach to automated recognition of gastric mucosal features. To further advance the rigor and clinical relevance of this work, I offer several observations that warrant attention.

First, the manuscript does not clarify the consistency of image annotation. The Kyoto Gastritis Score involves subjective judgments across five key endoscopic findings. However, details regarding the number of annotators, their expertise, and interrater reliability metrics such as Cohen’s or Fleiss’ kappa are absent. Without assessing annotation agreement, the risk of label noise remains [2,3], potentially undermining model robustness and generalizability. I encourage incorporation of standardized multi-expert annotation protocols and explicit reliability assessment in future studies.

Second, the clinical diversity of the patient population behind the image data set is not sufficiently described. Important factors like age, sex, *Helicobacter pylori* status, and disease stage can influence gastric mucosal appearance and thus model performance [4]. Lack of transparency about these variables limits interpretation and raises concerns about selection bias. Comprehensive reporting and subgroup analyses would enhance understanding of the model’s applicability across varied clinical contexts.

Third, the model treats the five Kyoto features as independent classification targets without accounting for their pathological interrelations. Features such as gastric atrophy and intestinal metaplasia often coexist, and exploiting these associations could improve diagnostic accuracy. Integrating multi-task or graph-based learning approaches to capture feature dependencies would strengthen both predictive power and biological interpretability.

Fourth, the paper omits discussion of calibration for probabilistic outputs and threshold selection for multi-label classification. In clinical settings, Ill-calibrated confidence scores and data-driven thresholding are critical to balance sensitivity and specificity, minimizing false positives and negatives [5]. Adopting calibration methods such as temperature scaling and systematic threshold optimization would improve the clinical utility of AI outputs.

Finally, the description of image preprocessing steps is limited. Variability in endoscopic equipment and acquisition conditions affect image characteristics significantly. Transparent and detailed reporting of preprocessing—such as color normalization, noise reduction, and resolution standardization—is essential to ensure model robustness across heterogeneous data sources.

Addressing these points will enhance the reliability, interpretability, and translational potential of AI models for gastric disease diagnosis. I encourage further research that incorporates rigorous annotation standards, clinical heterogeneity, feature dependency modeling, calibrated outputs, and comprehensive preprocessing. These refinements will help realize the full promise of AI-assisted endoscopy in routine clinical practice.

## Data Availability

Data sharing is not applicable to this article as no datasets were generated during current study.
